# Promoting the Selectivity of Pt/m-ZrO_2_ Ethanol Steam Reforming Catalysts with K and Rb Dopants

**DOI:** 10.3390/nano11092233

**Published:** 2021-08-29

**Authors:** Michela Martinelli, Richard Garcia, Caleb D. Watson, Donald C. Cronauer, A. Jeremy Kropf, Gary Jacobs

**Affiliations:** 1 Center for Applied Energy Research, University of Kentucky, 2540 Research Park Drive, Lexington, KY 40511, USA; michela.martinelli@uky.edu; 2 Department of Biomedical Engineering and Chemical Engineering, The University of Texas at San Antonio, 1 UTSA Circle, San Antonio, TX 78249, USA; g123richard@gmail.com (R.G.); caleb.watson378@gmail.com (C.D.W.); 3 Argonne National Laboratory, Lemont, IL 60439, USA; dccronauer@anl.gov (D.C.C.); kropf@anl.gov (A.J.K.); 4 Department of Mechanical Engineering, The University of Texas at San Antonio, 1 UTSA Circle, San Antonio, TX 78249, USA

**Keywords:** ethanol steam reforming, potassium, rubidium, basicity, zirconia, XANES, DRIFTS

## Abstract

The ethanol steam reforming reaction (ESR) was investigated on unpromoted and potassium- and rubidium-promoted monoclinic zirconia-supported platinum (Pt/m-ZrO_2_) catalysts. Evidence from in situ diffuse reflectance infrared Fourier transform spectroscopy (DRIFTS) characterization indicates that ethanol dissociates to ethoxy species, which undergo oxidative dehydrogenation to acetate followed by acetate decomposition. The acetate decomposition pathway depends on catalyst composition. The decarboxylation pathway tends to produce higher overall hydrogen selectivity and is the most favored route at high alkali loading (2.55 wt.% K and higher or 4.25 wt.% Rb and higher). On the other hand, decarbonylation is a significant route for the undoped catalyst or when a low alkali loading (e.g., 0.85% K or 0.93% Rb) is used, thus lowering the overall H_2_ selectivity of the process. Results of in situ DRIFTS and the temperature-programmed reaction of ESR show that alkali doping promotes forward acetate decomposition while exposed metallic sites tend to facilitate decarbonylation. In previous work, 1.8 wt.% Na was found to hinder decarbonylation completely. Due to the fact that 1.8 wt.% Na is atomically equivalent to 3.1 wt.% K and 6.7 wt.% Rb, the results show that less K (2.55% K) or Rb (4.25% Rb) is needed to suppress decarbonylation; that is, more basic cations are more efficient promoters for improving the overall hydrogen selectivity of the ESR process.

## 1. Introduction

In recent decades, the catalytic steam reforming of hydrocarbons such as natural gas has been the most economically competitive method to produce hydrogen in the chemical industry. However, this method is not sustainable as the feedstock is a fossil source, and significant amounts of CO_2_ are produced in the process. Thus, the development of new sustainable reforming technologies from renewable feedstocks (e.g., biomass-derived oxygenates) is necessary for reducing net greenhouse gas emissions [1]. In this scenario, researchers are focusing on several renewable feedstocks such as ethanol, polyols, and dimethyl ether [2,3,4,5]. Among these renewable feedstocks, bio-ethanol is very attractive because of its favorable hydrogen content, wide abundance, low toxicity, and ability to be easily stored for transportation and portable power [3]. Moreover, while ethanol production (e.g., from sugar cane or corn) currently competes with food production, cellulosic ethanol is currently under development. The overall ethanol steam reforming (ESR) reaction, which occurs at 350–650 °C, can be summarized by Equation (1):

C_2_H_5_OH + 3H_2_O → 2CO_2_ + 6H_2_ (1)

Acetaldehyde, ethylene, and methane are formed during ESR. However, the concentration of these species must be minimized to achieve higher efficiency of hydrogen production and avoid carbon formation and consequently catalyst deactivation [6]. These intermediates and byproducts can be converted via different pathways, depending on catalyst structure as well as the conditions used in the reactor [3].

Ethanol steam reforming catalysts are often transition metals such as copper, cobalt, and nickel, or noble metals such as platinum, palladium, rhodium, gold, or ruthenium; combinations of metals have also been used [7,8]. Among the transition metals, nickel and cobalt are the most common. However, catalyst deactivation by coke deposition is a major issue for these catalysts [6,8,9,10,11,12,13,14]. Noble metals such as Pt and Rh have better resistance to coke deposition as well as high activity, but they are much higher in cost [8,15,16,17,18]. These metals are typically supported on basic, acidic, or inert supports [7]. Oxides that form surface defects through partial reduction (e.g., ceria, zirconia, ceria–zirconia mixtures, and metal-promoted ceria catalysts) have also been investigated because of their high oxygen mobility, their ability to dissociate water or ROH (i.e., where R is an alkyl group - methanol, ethanol, etc.), and their capability to shuttle O-bound intermediates on the catalyst surface [19,20]. Ciambelli et al. [21] observed that Pt/CeO_2_ exhibited higher activity as compared to Pt/Al_2_O_3_ for ESR in the temperature range of 300–450 °C. He et al. [22] obtained similar findings. The high activity of Pt/CeO_2_ was due in part to the ability of Pt to facilitate the formation of defect sites on the partially reducible oxide support. ROH molecules can then dissociate on these defect sites [23], which is comparable to the adsorption of H_2_O at defect sites that result in the formation of bridging OH groups [24,25,26].

Recently, the alkali promotion of noble or transition metal catalysts has been investigated. Alkali doping can impact many catalyst properties, including activity, stability, resistance to coke formation, selectivity, and surface acidity/basicity [6,27,28,29,30,31,32,33,34,35,36,37,38,39,40,41,42]. The effect of alkali on nickel-based catalysts is contradictory. Frusteri et al. [33,35] found that Li and Na adversely affect the nickel dispersion, whereas they improve the extent of reduction of nickel. In contrast, no effect on either Ni dispersion or catalyst morphology was detected for K-promoted catalysts. Moreover, the authors examined the influence of alkali loading on catalyst performance. Lithium and potassium enhanced catalyst stability mainly by depressing nickel sintering, whereas carbon laydown did not seem to be influenced by adding K. However, a different effect of potassium was observed by Slowik et al. [6]. In that case, potassium promotion of Ni/CeO_2_ was not found to protect the catalyst against the formation of carbon deposits and did not improve stability during ESR. 

Improvements in stability/activity were reported for cobalt-based catalysts, and this is mainly related to the inhibition of carbon deposition [27,28,29,30,31,38]. Recently, Grzybek et al. [27] studied the alkali surface state (location, dynamics) by Species Resolved Thermal Alkali Desorption (SR-TAD). Movement of potassium from cobalt to alumina was observed during both activation and ESR. This phenomenon stabilizes small cobalt crystallites by hindering their detachment from the catalyst surface, which would otherwise result in encapsulation by the growing carbonaceous deposit. Furthermore, Grzybek et al. [28] found that potassium loadings from 0.1 to 4 wt.% improve the activity of their catalyst by enhancing C–C bond scission, but 0.3 wt.% is the optimal loading to maximize the selectivity to H_2_ and CO_2_, the most desirable products. The beneficial effect of K can also be related to improvement in reduction of Co^2+^ to Co^0^, the stabilization of acetate species, and the suppression of methane formation [31].

To our knowledge, not many studies are available on the effect of alkali for noble metal catalysts, especially at low temperatures [34,40,41,42]. Low potassium loading (0.2%) was found to decrease the initial conversion but improves the stability for Rh/CeO_2_-ZrO_2_. At higher loading (5%), the catalyst activity is negligible [42]. Dömök et al. explored doping potassium to Pt/Al_2_O_3_ catalyst, and they found that increasing potassium content progressively decreases the ethanol conversion and changes the product distribution toward a higher selectivity to CH_4_ and CO_2_ as compared to 1% Pt/Al_2_O_3_ [34]. Furthermore, potassium destabilizes adsorbed acetate, promoting its decomposition to CO_2_ and CH_4_ at a lower temperature; on the other hand, acetate species were more stable on the undoped catalyst, decomposing at ~420 °C [34]. 

In our prior study of Na-doping to Pt/ZrO_2_ [40,41], a similar trend was obtained, as acetate decomposed at a lower temperature (100–150 °C) when 1.8–2.5 wt.% Na was added to the formulation as compared to the undoped catalyst. Our focus is on the low-temperature conversion of ethanol with steam to H_2_, CO_2_, and CH_4_, with the latter being reformed in a conventional methane reforming (e.g., autothermal reforming). With this decarboxylation route Equation (1), which involves the steps below Equations (2) and (3), higher hydrogen selectivity is expected as compared to the decarbonylation route Equation (4):

Decarboxylation pathway:

C_2_H_5_OH + H_2_O → 2H_2_ + CH_4_ + CO_2_ (2)

CH_4_ + 2H_2_O → 4H_2_ + CO_2_ (3)

Decarbonylation pathway

C_2_H_5_OH + H_2_O → 4H_2_ + 2CO (4)

In that work, DRIFTS experiments revealed that the acetate decomposition pathway depends on the Na loading. Forward direct acetate decomposition to CH_4_ and carbonate Equation (2) is the most favorable pathway at high sodium loading (1.8 or 2.5 wt.%), whereas the unselective decarbonylation route occurs for the unpromoted catalyst or at low sodium loading (0.5 wt.%) and is promoted by metallic sites.

The question remains as to whether more basic alkali metals might further improve the decarboxylation selectivity over that of decarbonylation during ESR. To that end, the effect of potassium and rubidium loading on the relative rates of decarboxylation/decarbonylation was investigated. Pt/m-ZrO_2_ catalyst was promoted by the following potassium loadings: 0% (reference), 0.85%, 1.70%, 2.55%, 3.40%, 4.25%, and 8.50 wt.%; whereas rubidium was added with the following loadings: 0.93%, 1.86%, 2.79%, 3.72%, 4.65%, 5.59%, and 9.29%. Atomically equivalent loadings allowed for some comparisons between the K- and Rb-promoted catalysts, as well as with catalysts prepared in our earlier study using Na as the dopant [40,41]. These systems were characterized by N_2_ physisorption, transmission electron microscopy (TEM), X-ray absorption near edge spectroscopy (XANES), extended X-ray absorption fine structure spectroscopy (EXAFS), hydrogen temperature programmed reduction (H_2_-TPR), temperature-programmed ESR, and DRIFTS. Catalyst activity and selectivity were measured at steady-state using a fixed bed tubular reactor.

## 2. Materials and Methods

### 2.1. Catalyst Preparation

Various potassium and rubidium loadings on 2% Pt/ZrO_2_ were prepared via incipient wetness impregnation (IWI). Firstly, monoclinic ZrO_2_ (Alfa Aesar, Haverhill, MA, USA) was impregnated by 2% Pt with an aqueous solution of tetraamine platinum (II) nitrate (Alfa Aesar, Haverhill, MA, USA) followed by drying and calcination at 350 °C (four hours, muffle furnace). Then, the appropriate amount of KNO_3_ (Alfa Aesar, Haverhill, MA, USA) or RbNO_3_ (Alfa Aesar, Haverhill, MA, USA) was added via IWI. Promoted catalysts were dried and recalcined using the same conditions.

### 2.2. Catalyst Characterization

Surface area and pore size were determined using a Micromeritics 3-Flex instrument (Micromeritics, Norcross, GA, USA). The BJH method was used to calculate the average pore diameter and specific volume. Samples were pre-treated at 160 °C at 50 mTorr for no less than 12 h. 

H_2_ temperature-programmed reduction (TPR) plots of the catalysts were obtained using an Altamira AMI-300R (Altamira, Pittsburgh, PA, USA) instrument employing a thermal conductivity detector (TCD). During experiments, approximately 200 mg of catalyst was loaded into the U-tube reactor, and then a mixture of 10% H_2_ in Ar (30 cm^3^/min) (Airgas, San Antonio, TX, USA) was flowed while the temperature was ramped from 50 to 1000 °C at a heating rate of 10 °C/min. 

Transmission electron microscopy (TEM) and scanning transmission microscopy (STEM) were conducted with an FEI Talos F200X scope (Thermo Fisher Scientific, Waltham, MA, USA) equipped with bright field (BF), dark-field (DF) 2, DF 4, and high-angle annular dark-field (HAADF) detectors. Imaging was performed using a field emission gun with an accelerating voltage of 200 kV and a high-speed Ceta 16M camera. The elemental distributions were determined via FEI super energy-dispersive X-ray spectroscopy (EDX) (Thermo Fisher Scientific, Waltham, MA, USA). Velox software was utilized to process the data. Before analysis, samples were first treated in H_2_ at 350 °C for 1 hour and then cooled to room temperature, followed by passivation with 1% O_2_ (balance N_2_). Then, the reduced/passivated samples were sonicated for 30 min in ethanol. A sample of this suspension was dropped onto a lacey carbon-covered Cu grid (300 mesh) and dried in air for 12 h.

Temperature-programmed reaction/desorption analyses were carried out with the Altamira AMI-300R unit. For each experiment, the catalyst was reduced at 300 °C using 10 cm^3^/min H_2_ (Airgas, San Antonio, TX, USA) and 20 cm^3^/min argon (Airgas, San Antonio, TX, USA) for 1 h. After cooling the catalyst to 50 °C in flowing Ar, ethanol was pumped at a rate of 100 mL/min across the catalyst for 10 min, and then Ar was flowed at 30 cm^3^/min for 20 min. Following this purge, water was bubbled in He at a rate of 30 cm^3^/min for 10 min, and then another purge in Ar was performed. Finally, Ar was flowed at 30 cm^3^/min while the temperature was increased to 700 °C. This allowed analysis of ESR under transient conditions. CH_4_ evolution was measured to examine the effect of K or Rb on C–C cleaving.

A Nicolet (Thermo Fisher, Waltham, MA, USA) iS-10 Fourier Transform infrared spectrometer, coupled with a Harrick Scientific (Pleasantville, New York, NY, USA) Praying Mantis accessory, was used for the temperature desorption/reaction experiments. The catalyst was reduced at 300 °C with a 1:1 mixture of H_2_:He at a flow rate of 200 cm^3^/min for 1 h and then cooled to 50 °C in hydrogen. Next, helium was used to bubble ethanol at a flow rate of 75 cm^3^/min for 15 min. Subsequently, He was bubbled through water (31 °C water bath), giving an H_2_O concentration of 4.4% with a flow rate of 75 cm^3^/min. Temperature was stepped in 50 °C increments from 50 °C to 500 °C. At each step, 512 scans were taken at a resolution of 4.

Temperature programmed reduction with X-ray absorption near edge spectroscopy (TPR-XANES) was performed at the Materials Research Collaborative Access Team (MR-CAT) beamline located at the Advanced Photon Source, Argonne National Laboratory. Sample amount was optimized for the platinum L_3_ edge. The quartz reactor was held in a clamshell furnace located on a positioning table, and the beam passed through six samples in a sequential manner with 20 μm accuracy for repeat scans. He was flowed through the catalysts for more than 5 min at a flow rate of 30 mL/min. Pure hydrogen was then passed through the sample at a flow rate of 30 mL/min, and a heating rate of 0.83 °C/min was started for the furnace to achieve 300 °C. After soaking at this temperature for 1 h, samples were cooled to room temperature and scanned for both the XANES and EXAFS regions at both the platinum L_3_ (11.564 keV) and L_2_ edges (13.273 keV), so that the L_3_-L_2_ edge difference procedure could be applied [43]. Spectra were recorded in transmission mode, and the respective metallic foil was measured in a concurrent manner for the purpose of energy calibration. The Pt L_3_ edge data were in the range of 11.400–12.700 keV, and Pt L_2_ edge data were in the range of 13.100–13.850 keV. Standard data reduction was conducted with WinXAS (Version 2.0, Thorsten Ressler, Berlin, Germany) [44], while fittings were carried out for EXAFS with Atoms (Copyright 2001, Department of Physics, University of Washington, Seattle, WA, USA) [45], FEFF8 (Version 8.20, Department of Physics, University of Washington, Seattle, WA, USA) [46], and FEFFIT (Copyright 2001, Department of Physics, University of Washington, Seattle, WA, USA) [46] software over Δk = 3–10 Å^−1^ and ΔR = 1.85–3.25 Å. A typical analysis included post-edge background subtraction (Victoreen function), pre-edge and post-edge background subtraction (degree 1 polynomials), normalization based on the edge jump, conversion to k-space with background subtraction using a cubic spline, and applying a Fourier transform of the χ(k) function to R-space.

### 2.3. Catalytic Activity

The activity of the catalysts was tested in a fixed bed reactor (stainless steel tubular reactor, I.D. 0.444 in.); more information on the experimental set-up is reported in our previous study [47]. Briefly, 80 mg of catalyst (63–106 µm) was diluted with 300 mg of SiO_2_ beads and activated using 100 cm^3^/min H_2_ at 350 °C for 1 h. Next, the temperature was cooled to 300 °C, and the gas was changed to a mixture containing 26.1% H_2_O, 2.9% C_2_H_5_OH (balance N_2_) at P = 1 atm, gas hourly space velocity (GHSV) = 190,560 Ncm^3^/min/g_cat_. The unpromoted catalyst was also tested at different GHSV in order to compare the selectivities among the catalysts at similar conversion. The products were then passed through a cold trap (held at 5 °C) to collect condensable compounds. The condensable products were analyzed by SRI (SRI Instruments, Torrance, CA, USA) 8610 GC equipped with HayeSep Q-column, whereas the gas products were analyzed by Inficon micro-GC Fusion equipped by molecular sieve, alumina, plot-u, and OV-1. Ethanol conversion Equation (5), carbon selectivity Equation (6), and H_2_ yield Equation (7) were calculated using the following formulas:

(5) $$\chi_{C_{2}H_{5}OH}=1-\frac{F_{C_{2}H_{5}OH}^{out}}{F_{C_{2}H_{5}OH}^{in}}$$

(6) $$S_{i}=\frac{n_{i}\cdot F_{i}^{out,\;prod}}{\sum_{i}n_{i}\cdot F_{i}^{out,prod}}$$

(7) $$H_{2}\;yield=\frac{F_{H2}^{out,\;prod}}{6\cdot F_{C2H5OH}^{in}}$$

where $F_{C_{2}H_{5}OH}^{in}$ is the molar feed rate of ethanol, $F_{C_{2}H_{5}OH}^{out}$ is the effluent molar flow rate, $n_{i}$ is carbon number, $F_{H2}^{out,\;prod}\;$ is the effluent molar flow rate of *H*_2_, and $F_{i}^{out,\;prod}$ is effluent molar flow rate of the C-containing species (*i* = CO, CO_2_, C_2_H_6_, C_2_H_4_, C_3_H_6_, C_2_H_4_O). 

## 3. Results and Discussion

Surface area and porosity results for un-promoted and K and Rb-promoted catalysts are provided in Table 1. Both K and Rb series showed that low alkali loadings caused a slight decrease in the surface area, whereas high loadings dramatically decreased it (i.e., below what is expected from the decrease due to the added mass of alkali). For example, the surface area dropped from 89.7 m^2^/g_cat_ (unpromoted) to 34.7 and 58.2 m^2^/g_cat_ for 8.5% K and 9.29% Rb, respectively. This decrease in the surface area suggests that pore blocking is more significant at high alkali loading. Increasing alkali doping progressively diminished the pore volume, but little impact was observed for the average pore diameter of the Rb-promoted catalyst. In contrast, above 2.55% K, the average diameter increased systematically, suggesting preferential blocking of narrower pores by K at higher loadings.

Estimated Pt diameter and dispersion obtained by EXAFS fittings are also reported in Table 1. Interestingly, the diameter of the Pt cluster increases with the alkali loading, and the effect is more pronounced for the K-promoted catalyst. This results in a decreasing trend in Pt dispersion, which drops from 88% and 94% for the unpromoted catalysts to 35% and 56% for 4.25% K-Pt/ZrO_2_ and 9.29% Rb-Pt/ZrO_2_, respectively.

**Figure 1:**
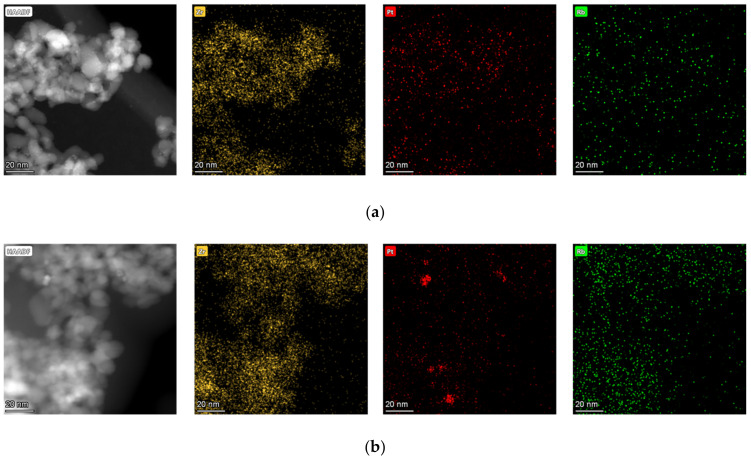
Transmission electron microscopy (TEM) and scanning transmission microscopy with energy-dispersive X-ray spectroscopy (STEM-EDX) images for the (**a**) 0.93% Rb-2% Pt/ZrO_2_ catalyst and (**b**) the 9.29% Rb-2% Pt/ZrO_2_ catalyst.

STEM-EDX images for Rb-promoted catalysts are shown in Figure 1. Both platinum and rubidium were well dispersed for 0.93% Rb-2% Pt/ZrO_2_, and all clusters observed were below 3 nm. By increasing the rubidium loading to 9.29%, STEM-EDX images (Figure 1, bottom) show rubidium was well dispersed, whereas platinum particles tended to form agglomerates of several Pt domains. Some agglomerates were on the order of 10 nm, while the domains were typically below 3 nm. The spatial distribution of Pt and Rb also suggests, especially in the case of 9.29% Rb, that there is a strong possibility of contact between the two elements.

**Figure 2:**
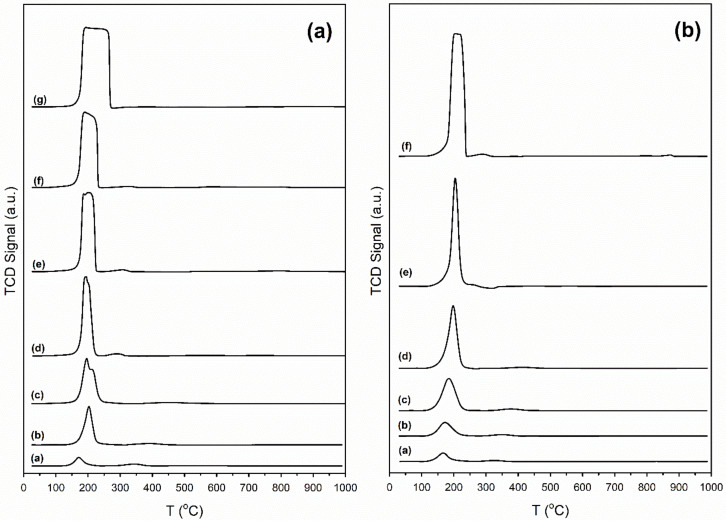
(**a**): TPR profiles for K-promoted catalysts: (a) 2% Pt/ZrO_2_, (b) 0.85% K-2% Pt/ZrO_2_, (c) 1.70% K-2% Pt/ZrO_2_, (d) 2.55% K-2% Pt/ZrO_2_, (e) 3.40% K-2% Pt/ZrO_2_, (f) 4.25% K-2% Pt/ZrO_2_, and (g) 8.50% K-2% Pt/ZrO_2_; (**b**): TPR profile for Rb-promoted catalysts: (h) 0.55% Rb-2% Pt/ZrO_2_, (i) 1.86% Rb-2% Pt/ZrO_2_, (j) 2.79% Rb-2% Pt/ZrO_2_, (k) 4.65% Rb-2% Pt/ZrO_2_, and (l) 9.29% Rb-2% Pt/ZrO_2._ Figure 2a reprinted from [48] with permission from Elsevier, copyright 2020. Figure 2b reprinted from [48] with permission from MDPI, copyright 2021.

Catalyst reducibility was investigated by TPR (Figure 2). The unpromoted Pt/zirconia catalyst had a small hydrogen uptake in the range of 150–200 °C, indicative of reduction of platinum oxide to metal, which occurs around 200 °C, and Pt-catalyzed defect formation [46]. Pt accelerates the decomposition of surface carbonates and facilitates the formation of oxygen vacancies and bridging OH groups [25,52,53]. The hydrogen uptake during TPR further increased by adding potassium or rubidium.

**Figure 3:**
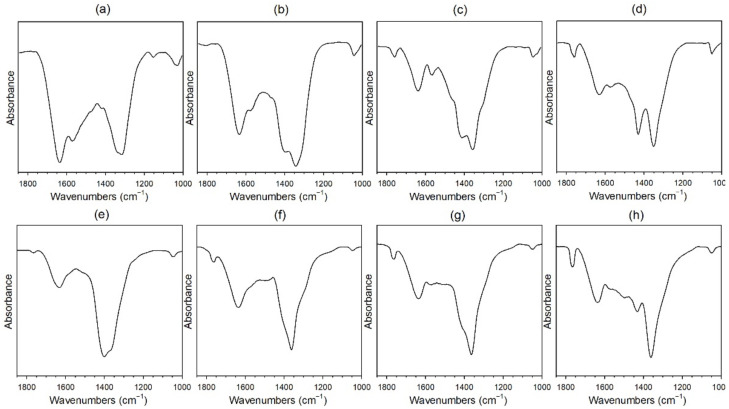
DRIFTS spectra of carbonate decomposition during activation for (**a**) 2% Pt/ZrO_2_, and the same doped with (**b**) 0.93% Rb, (**c**) 4.65% Rb, (**d**) 9.29% Rb, (**e**) 0.85% K, (**f**) 2.55% K, (**g**) 4.25% K, (**h**) 8.50% K. Figure 3a–d reprinted from [49] with permission from MDPI, copyright 2021. Figure 3e,f reprinted from [48] with permission from Elsevier, copyright 2020.

Temperature programmed reduction with mass spectrometry (TPR-MS) spectra for K and Rb-promoted catalysts, reported in our previous works [48,49], showed that the evolution of carbon monoxide occurs concurrently with hydrogen uptake, especially for high alkali loading. This indicates the presence of surface carbonates on the catalyst before H_2_ activation, as previously noted [52,53]. Doping with K or Rb increases surface basicity, such that more carbonate (i.e., adsorbed carbon dioxide—which is acidic) decomposes from the catalyst as the alkali loading is increased. Decomposition of these surface carbonates through Pt-assisted decarbonylation is easily monitored by in situ DRIFTS (Figure 3) with the generation of CO via Pt carbonyl species.

**Figure 4:**
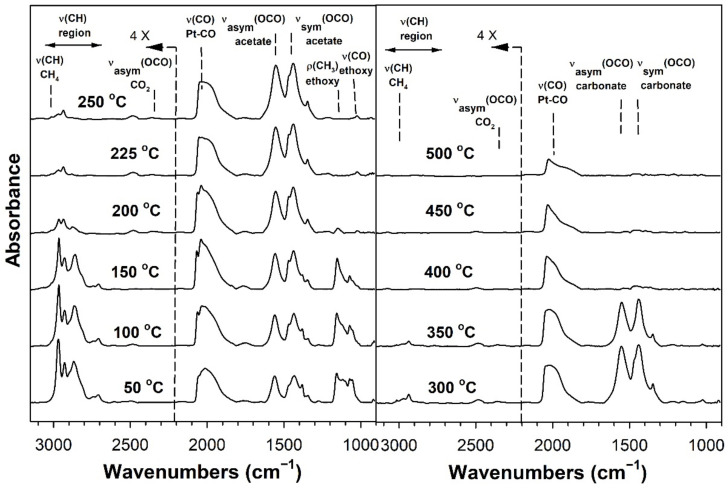
DRIFTS spectra of transient ESR over 2% Pt/m-ZrO_2_ (K-series).

**Figure 5:**
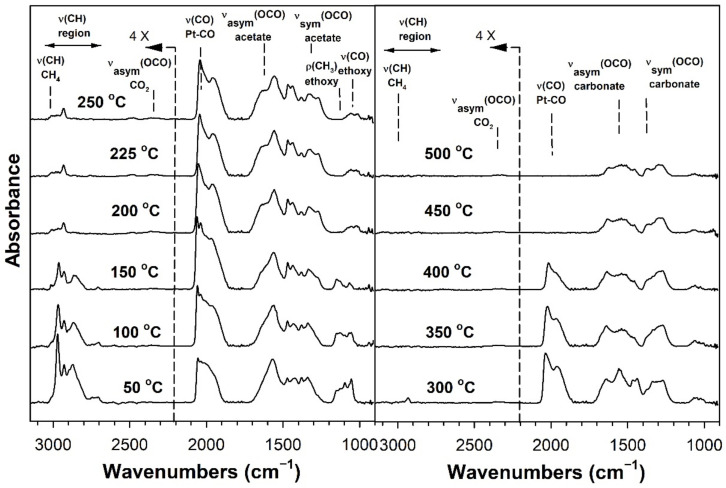
DRIFTS spectra of transient ESR over 0.85% K-2% Pt/m-ZrO_2_.

**Figure 6:**
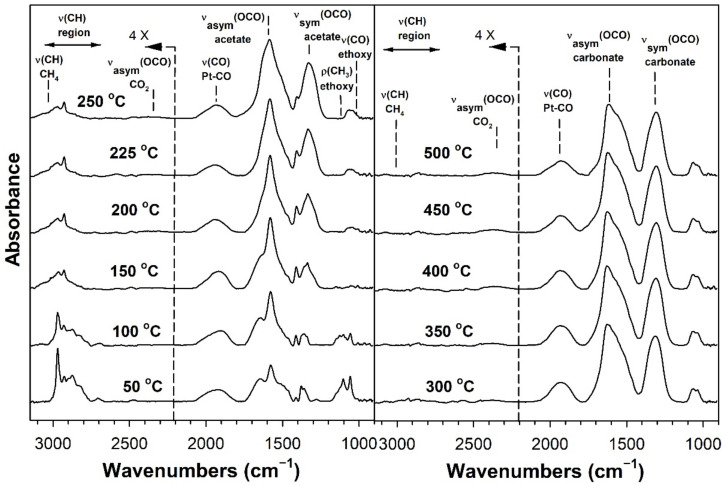
DRIFTS spectra of transient ESR over 2.55% K-2% Pt/m-ZrO_2_.

**Figure 7:**
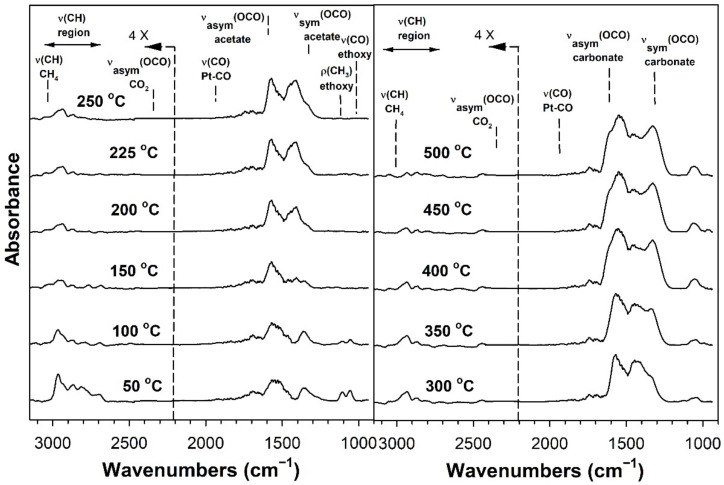
DRIFTS spectra of transient ESR over 4.25% K-2% Pt/m-ZrO_2_.

**Figure 8:**
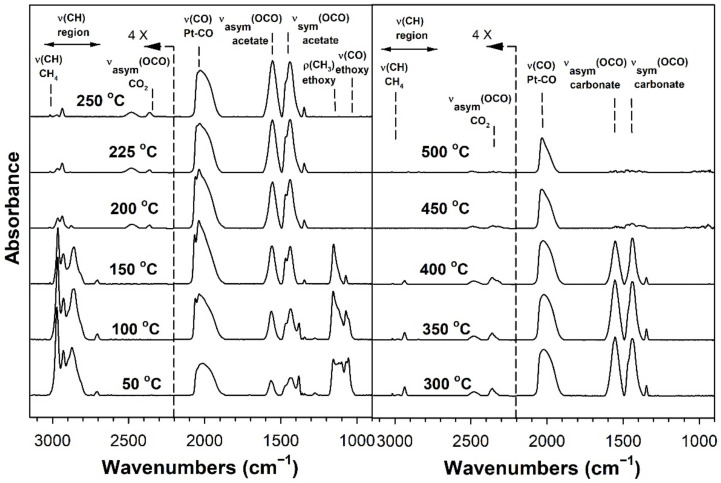
DRIFTS spectra of transient ESR over 2% Pt/m-ZrO_2_ (Rb-series).

**Figure 9:**
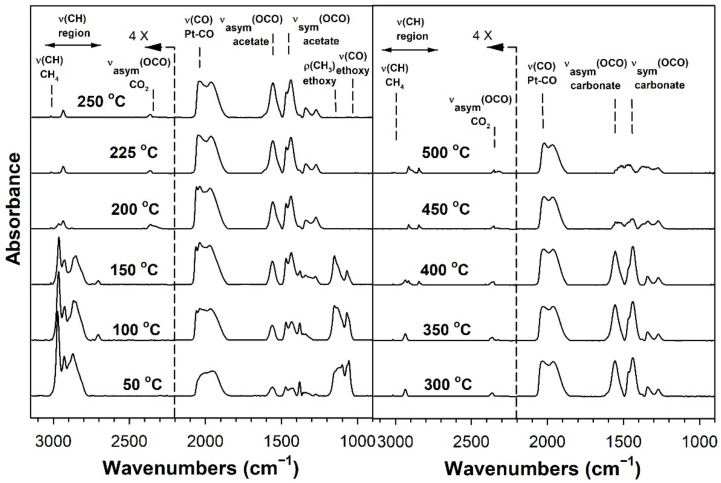
DRIFTS spectra of transient ESR over 0.93% Rb-2% Pt/m-ZrO_2_.

**Figure 10:**
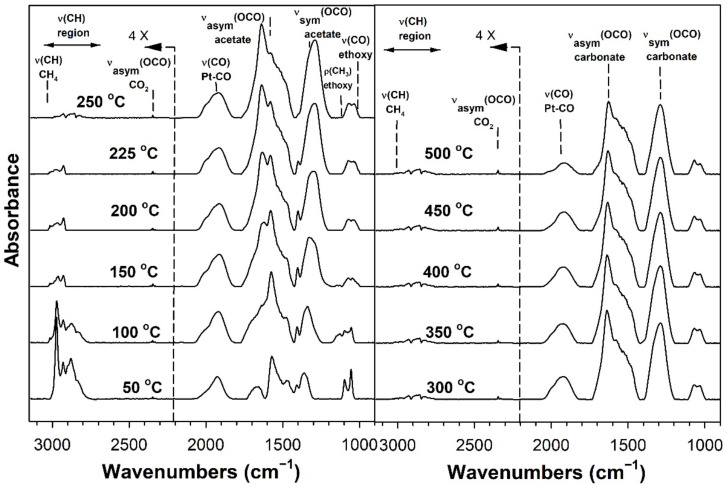
DRIFTS spectra of transient ESR over 4.65% Rb-2% Pt/m-ZrO_2_.

**Figure 11:**
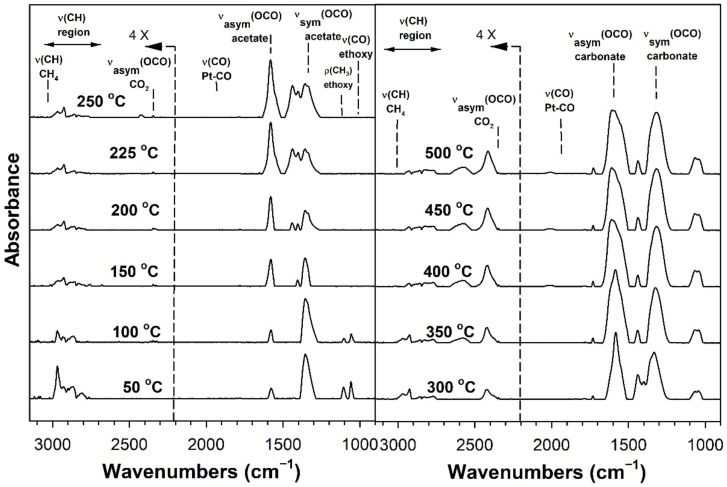
DRIFTS spectra of transient ESR over 9.29% Rb-2% Pt/m-ZrO_2_.

Table 2 provides assignments from the open literature regarding adsorbed species during ESR. Ethanol adsorption during DRIFTS was found to produce two bands in the range 1000–1200 cm^−1^ (Figure 4, Figure 5, Figure 6 and Figure 7 for K series and Figure 8, Figure 9, Figure 10 and Figure 11 for Rb series), which are assigned to ethoxy species. These species are formed by the dissociative adsorption of ethanol on the catalyst surface [54,55,56]. Type II ethoxy species located at surface defects on zirconia exhibit a low wavenumber ν(CO) band at ~1050 cm^−1^, while Type I ethoxy species are associated with unreduced sites on zirconia and positioned at higher wavenumbers [23]. Acetate produced several observable bands: symmetric υ(OCO) stretching (1300 cm^−1^), asymmetric υ(OCO) stretching (1510 cm^−1^), and υ(C–H) stretching bands (2700–3100 cm^−1^). These assignments were confirmed by comparing our results with the literature [2]. The observed formation of acetate likely suggests that ethoxy species underwent oxidative dehydrogenation.

DRIFTS of transient ESR was conducted on unpromoted, K-promoted, and Rb-promoted catalysts to shed further light on the possible mechanism (Figure 4, Figure 5, Figure 6, Figure 7, Figure 8, Figure 9, Figure 10 and Figure 11). Bands of ethoxy species, formed from dissociative adsorption of ethanol, were observed in the range of 50–150 °C for the unpromoted catalyst (Figure 4 and Figure 8 for K-doped and Rb-doped series, respectively). Increasing the temperature further, the surface concentration of ethoxy species decreased until they were virtually completely decomposed, while, simultaneously, there was a concomitant increase in the intensity of bands assigned to acetate; this suggests that ethoxy species underwent oxidative dehydrogenation to acetate—during this stage, CO_2_ gas was not produced. At the same time, the magnitude of the Pt-CO band increased, suggesting that decarbonylation occurred to a certain extent. After the amount of acetate attained a maximum of approximately 250–300 °C, further steam reforming afforded CH_4_ and CO_2_ in addition to CO. The detection of CH_4_ and CO_2_ is consistent with the forward decomposition of acetate. The acetate species nearly completely decomposed by 400 °C.

The effect of alkali promotion was also explored by conducting DRIFTS and varying the K loading (Figure 5, Figure 6 and Figure 7) and Rb loading (Figure 9, Figure 10 and Figure 11). DRIFTS revealed that the ethoxy intermediate reacted most rapidly on the 2.55% K- and 4.65% Rb-doped catalyst, evidenced by the fact that it was entirely converted to acetate by 150 °C through oxidative dehydrogenation. In contrast, at low-potassium (0.85%) or -rubidium (0.93%) loading, the ethoxy species exhibited greater stability, as they had significantly decomposed by only 200 °C, very similar to what was observed in the case of the undoped 2% Pt/m-ZrO_2_ catalyst. The acetate species is observed for all the potassium or rubidium loadings, suggesting it is a likely key intermediate during ESR. Once acetate is formed, however, DRIFTS results showed some differences among catalysts in terms of the selectivity of acetate decomposition, which depended on potassium or rubidium loading. The Pt–carbonyl bands for undoped, 0.85% K, and 0.93% Rb catalysts were at higher wavenumbers and at a significantly increased intensity, reaching maxima at 150–200 °C for the undoped, 150 °C for the 0.85% K doped, and 150–200 °C for the 0.93% Rb catalysts, respectively. This is reasonable to expect since acetate decomposes through different pathways depending on the level of alkali promotion. DRIFTS results suggest that acetate decarboxylation is preferred at high K (2.55% K and 4.25% K) or Rb (4.65% and 9.29% Rb) loading, while the decarbonylation is more favored on the unpromoted catalyst and lower loading alkali-doped catalysts (0.85% K and 0.93% Rb). The dependence of the two different decomposition pathways on alkali loading was also observed for the analogous Na-doped system for both ESR [40,41] and methanol steam reforming [57]. In the latter case, the analogous formate intermediate species were observed (formed from oxidative dehydrogenation of methoxy species), which decarboxylated at 2.5% Na loading, whereas it decarbonylated at low Na loading (i.e., 0.25%, 0.5%, 1%). This decarboxylation pathway enhanced CO_2_ selectivity and CO conversion, making 1.8% Na-2.5% Na the optimum loading range for H_2_ production.

DRIFTS results showed that acetate species decomposed at a lower temperature for the 2.55% K-doped catalyst and 4.65% Rb-doped catalyst as compared to the unpromoted catalysts, as a significant band for CH_4_ was detected at 150 °C (with a slight signal even at 100 °C), while a less intense band for CH_4_ was observed at 200 °C for the unpromoted catalysts (with a slight signal even detected at 150 °C). Thus, the acetate C–C bond breaks at lower temperatures for the K- and Rb-promoted catalysts.

This bond is analogous to the formate C–H band that is seen during water–gas shift or methanol steam reforming. In our prior WGS investigations, increasing Na, K, Rb, or Cs loading shifted the ν(CH) band to lower wavenumbers. At the same time, the difference between the ν(OCO) bands for asymmetric and symmetric stretching increased with alkali loading. This suggests that changes in surface basicity may be responsible for the CH bond weakening of formate. In the case of ESR, C–C bond weakening is not easily measured. However, we indeed see an increase in the wavenumber difference between the ν(OCO) bands for asymmetric and symmetric stretching. From Table 2, they are: 0% K, ∆ = 117 cm^−1^; 0.85% K, ∆ = 120 cm^−1^; 2.55% K, ∆ = 172 cm^−1^; 4.25% K, ∆ = 172 cm^−1^; 0% Rb, ∆ = 115 cm^−1^; 0.93% Rb, ∆ = 119 cm^−1^; 4.65% Rb, ∆ = 175 cm^−1^; and 9.29% Rb, ∆ = 176 cm^−1^. In all cases, the results suggest that the alkali promotes the weakening of the respective bond, emphasizing once again the analogous nature of the two alkali-doped catalyst systems. At 4.25% K or 9.29% Rb doping level, the catalyst surface was significantly blocked with the alkali, thereby creating a bottleneck for the formation of, and subsequent decomposition of, the intermediates. In addition to the CO_2_ that evolved, residual carbonates are clearly observed on the catalysts with K or Rb, as bands such as at 1620, 1574–1439 cm^−1^, and ~1315 cm^−1^ O–C–O stretching modes of carbonates [58].

Interestingly, the optimum K loading (2.55%) corresponds to a very similar weight percent as the optimal Na loading (2.5%) from prior work [41], meaning that the optimal K loading occurred at 60% of the optimal Na loading atomically. Some works have shown that at excessive alkali loadings on Pt/ZrO_2_, the surface of the Pt nanoparticles is blocked, inhibiting the role of Pt in hydrogen transfer reactions during LT-WGS [59,60]. This effect provides a reasonable explanation for the results obtained, given that K^+^ is considerably larger than Na^+^; the surface of the Pt nanoparticles becomes covered by the alkali metal at considerably lower atomic loadings for potassium-promoted catalysts. Additionally, it has been shown that at high alkali loadings, catalyst basicity promotes the formation of the carbonate intermediate, which is the precursor to CO_2_ formation; however, it was also found to impede CO_2_ liberation during LT-WGS [60,61]. This may be due to higher basicity since CO_2_ is an acidic molecule that is therefore adsorbed more strongly and/or it may be due to the fact that the alkali obstructs the metallic function, which is known to facilitate carbonate decomposition. Due to the fact that potassium is a more basic promoter than sodium, one would expect this effect to cause CO_2_ liberation to be significantly hindered for potassium relative to sodium. However, potassium is able to achieve similar promotion to sodium, and it does so at a lower atomic loading; this may be due to its lower electronegativity.

DRIFTS experiments showed that the optimal loading for the Rb-promoted catalyst is 4.65%, which is 80% and 50% of the optimal K and Na atomically loading, respectively. This trend suggests that a lower alkali atomically loading is required for the C–C bond scission of intermediate acetate when the alkali electronegativity decreases. Indeed, the electronegativity of Rb is 0.706 compared to 0.869 for Na and 0.734 for K [62]. A similar trend also was observed in our previous work [48]. Decreasing the electronegativity, the ν(C–H) formate band progressively shifted to lower wavenumbers, which was associated with a faster formate decomposition in the presence of steam [59,63,64].

**Figure 12:**
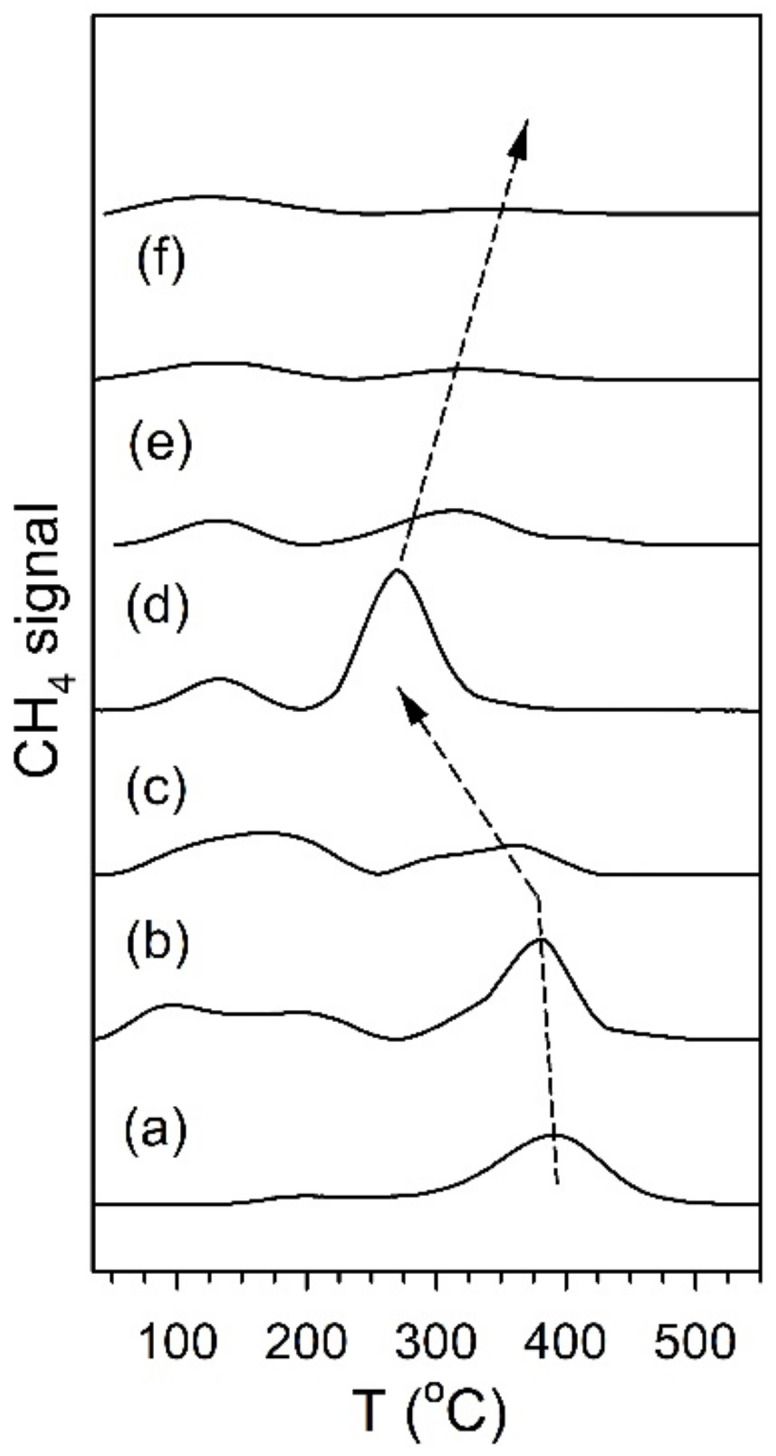
TPD-MS of ethanol steam reforming over (a) 2% Pt/ZrO_2_, (b) 0.85% K-2% Pt/ZrO_2_, (c) 1.70% K-2% Pt/ZrO_2_, (d) 2.55% K-2% Pt/ZrO_2_, (e) 3.40% K-2% Pt/ZrO_2_, (f) 4.25% K-2% Pt/ZrO_2_, and (g) 8.50% K-2% Pt/ZrO_2._

**Figure 13:**
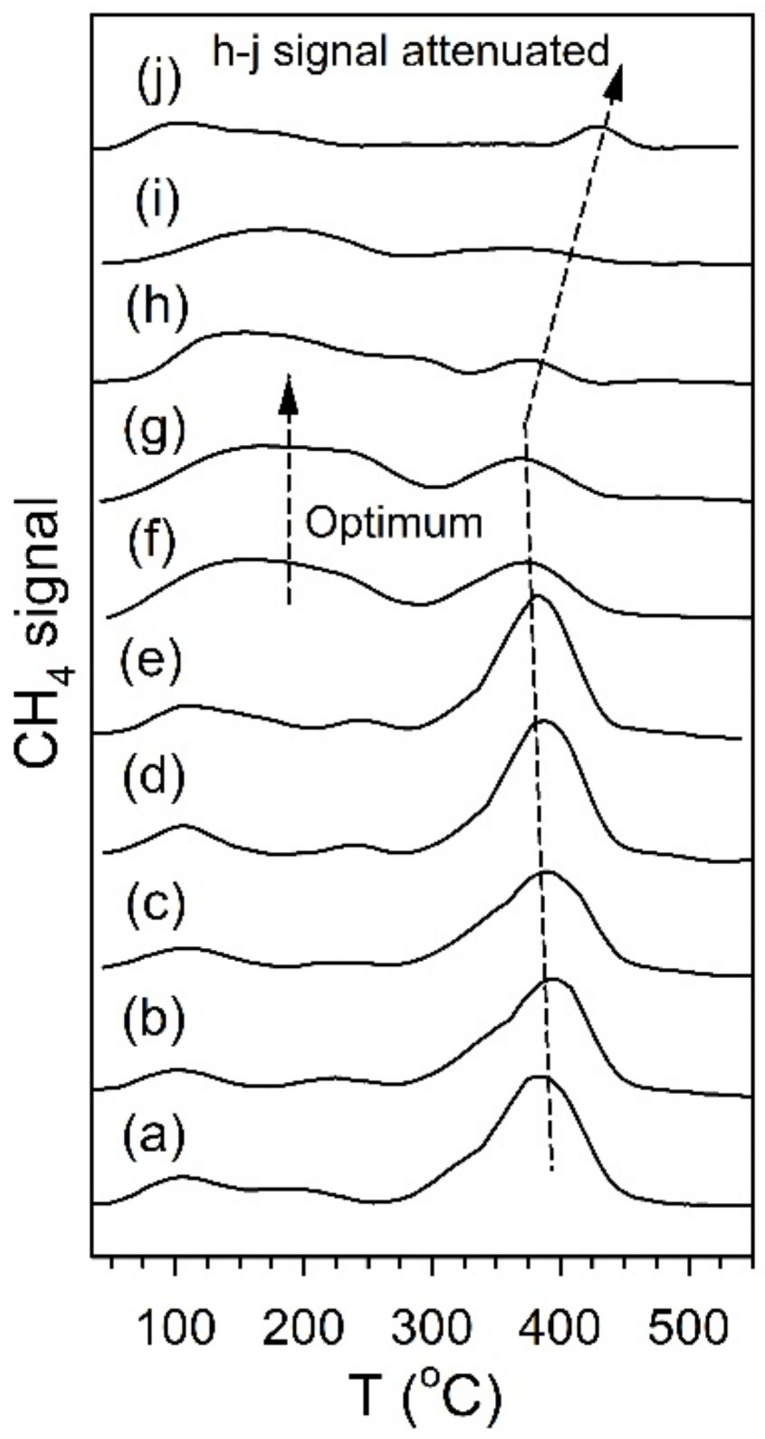
TPD-MS of ethanol steam reforming over (a) 2% Pt/ZrO_2_, (b) 0.37% Rb-2% Pt/ZrO_2_, (c) 0.74% Rb-2% Pt/ZrO_2_, (d) 0.93% Rb-2% Pt/ZrO_2_, (e) 1.86% Rb-2% Pt/ZrO_2_, (f) 2.79% Rb-2% Pt/ZrO_2_, (g) 3.72% Rb-2% Pt/ZrO_2,_ (h) 4.65% Rb-2% Pt/ZrO_2_, (i) 5.58% Rb-2% Pt/ZrO_2_, (j) 9.29% Rb-2% Pt-ZrO_2_.

**Figure 14:**
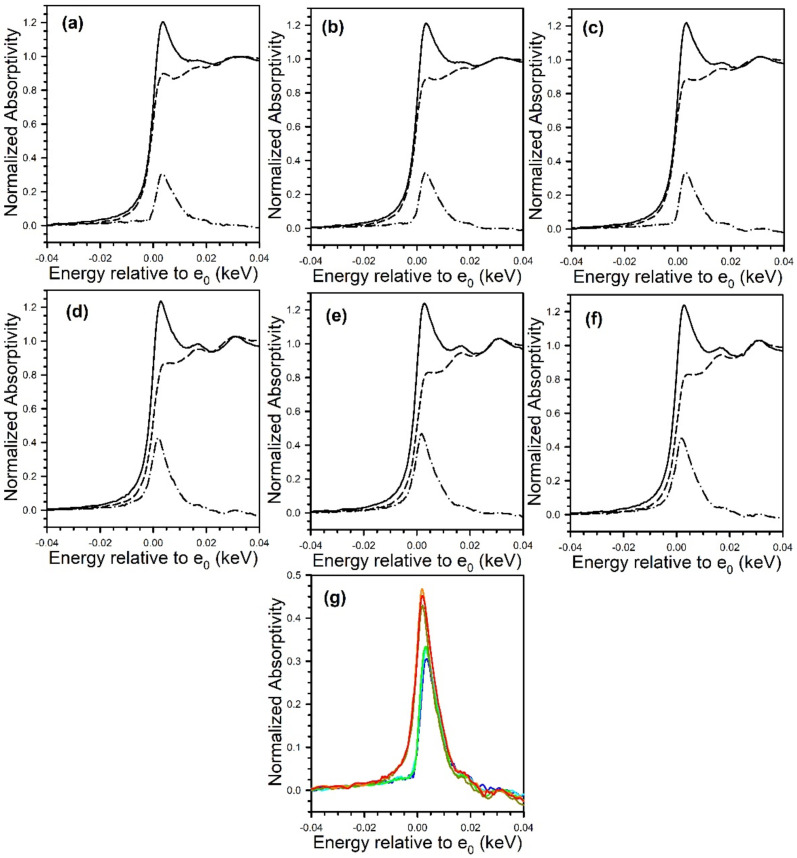
XANES and L_3_-L_2_ XANES difference spectra at the (dashed line) Pt L_2_ edge and (solid line) Pt L_3_ edge following reduction in pure hydrogen and cooling to ambient temperature, including 2% Pt/ZrO_2_ with: (**a**, blue) 0% K; (**b**, cyan) 0.85% K; (**c**, green) 1.70% K; (**d**, dark yellow) 2.55% K; (**e**, orange) 3.40% K; and (**f**, red) 4.25% K. (**g**) Overlays of L_3_-L_2_ difference spectra, showing an increase in intensity with K loading. No evidence for e^-^ transfer to Pt from K^+^ was found.

**Figure 15:**
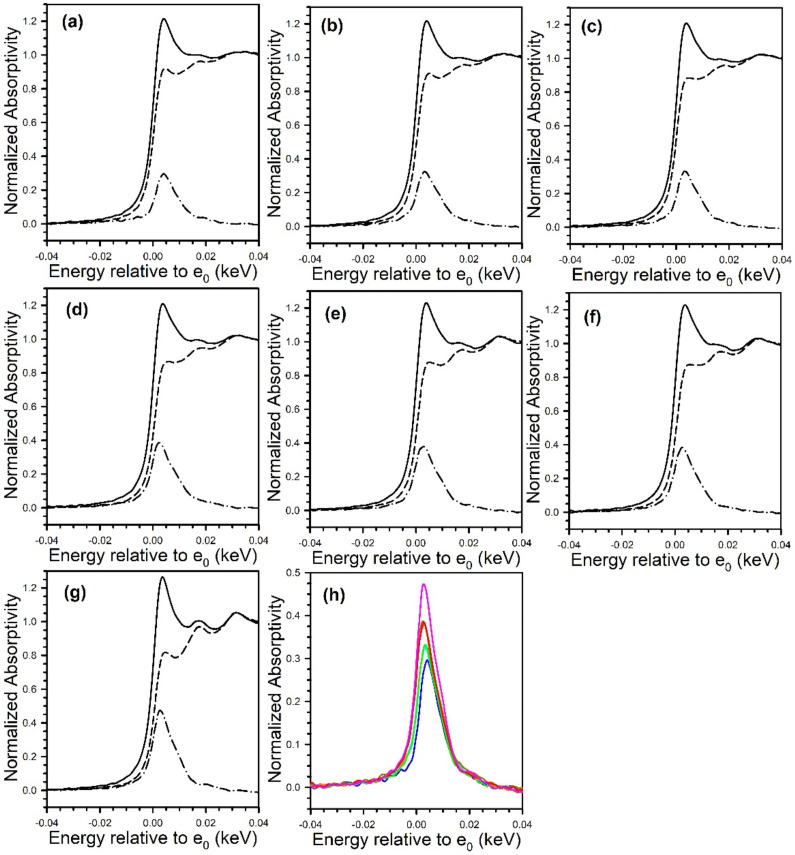
XANES spectra at the Pt (solid line) L_3_ edge and (dashed) line L_2_ edge, as well as (dash-dotted line) the L_3_-L_2_ difference spectra of (**a**, blue) 2% Pt/ZrO_2_, (**b**, cyan) 0.93% Rb-2% Pt/ZrO_2_, (**c**, green) 1.86% Rb-2% Pt/ZrO_2_, (**d**, dark yellow) 2.79% Rb-2% Pt/ZrO_2_, (**e**, orange) 4.65% Rb-2% Pt/ZrO_2_, (**f**, red) 5.58% Rb-2% Pt/ZrO_2_, and (**g**, pink) 9.3% Rb-2% Pt/ZrO_2_. (**h**) Overlays of L_3_ 2212 L_2_ difference spectra, showing an increase in intensity with Rb loading. No evidence for e^-^ transfer to Pt from Rb^+^ was found, which should result in an opposite trend. Figure 15 reprinted from [49] with permission from MDPI, copyright 2021.

**Figure 16:**
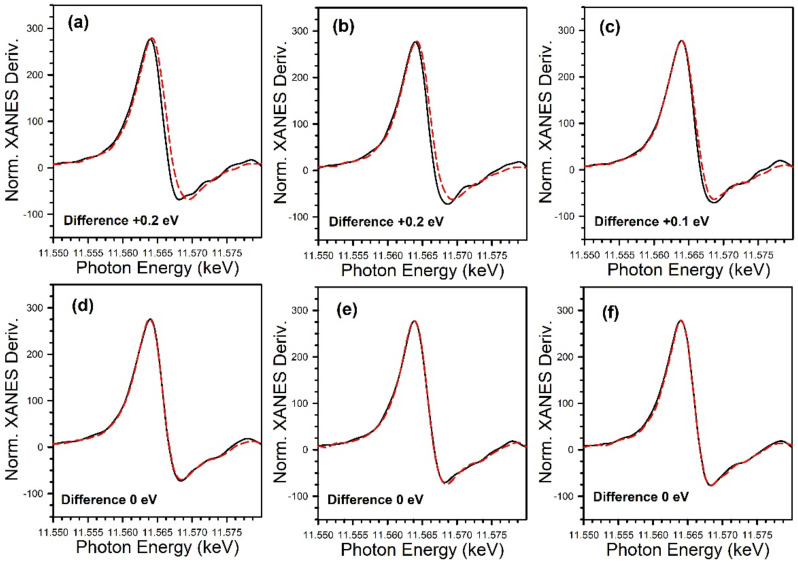
XANES derivative spectra at the Pt L_3_ edge of (solid line) the Pt^0^ foil and (red line) the catalysts following reduction in pure hydrogen, including 2% Pt/ZrO_2_ with: (**a**) 0% K; (**b**) 0.85% K; (**c**) 1.70% K; (**d**) 2.55% K, (**e**) 3.40% K; and (**f**) 4.25% K. No evidence for e^-^ transfer to Pt from K^+^ was found.

**Figure 17:**
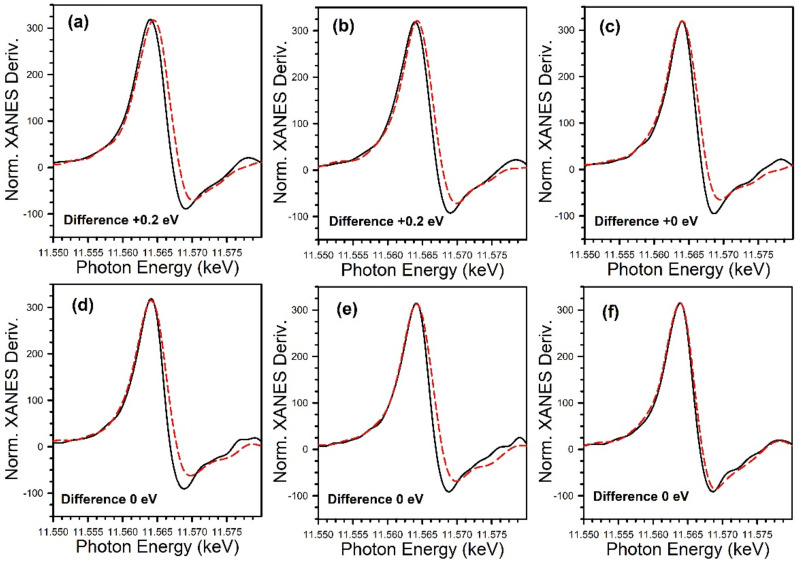
XANES derivative spectra at the Pt L_3_ edge of (solid line) the Pt^0^ foil and (red line) the catalysts following reduction in pure hydrogen, including 2% Pt/ZrO_2_ with: (**a**) 0% Rb, (**b**) 0.93% Rb, (**c**) 1.86% Rb, (**d**) 2.79% Rb, (**e**) 4.65% Rb, and (**f**) 9.3% Rb-2% Pt/ZrO_2_. No evidence for e^-^ transfer to Pt from Rb^+^ was found.

Figure 12 and Figure 13 show the TPD-MS profiles for methane over the K and Rb series of catalysts, respectively. The main peak for the 2% Pt/ZrO_2_ catalyst is at 391 °C, with a very minor peak at 200 °C. The position of the main peak did not change to a significant degree for the 0.85% K- and 1.70% K-doped catalysts, with peaks at 383 °C and 360 °C, respectively; minor peaks at low temperature were also observed at 100–190 °C and 115–170 °C. However, a significant shift to lower temperature occurred once the dopant loading reached 2.55% K, where the temperature was 270 °C for the main peak, with a low-temperature peak at 130 °C. At 3.40% K, 4.25% K, and 8.50% K doping levels, the main peak decreased in intensity and shifted to higher temperatures of 314 °C, 327 °C, and 345 °C, respectively, while the low-temperature peak remained at a similar temperature. The results indicate that cleaving of the C–C bond of acetate species during ESR is not only promoted by alkali addition but also that the K-doping loading is close to the optimum at 2.55% K. For the Rb series, increasing Rb loading to 1.86% Rb decreases the main CH_4_ evolution signal from 391 °C to 384 °C. In the range of 2.79% Rb–4.65% Rb, the peak at 370 °C is attenuated, and a new low-temperature peak at 160–180 °C emerges. At 5.58% and 9.29% Rb loadings, the CH_4_ signal is attenuated overall, and by 9.3% Rb, the signal of the higher temperature peak has increased to 430 °C due to blocking of the catalyst surface by excessive alkali. DRIFTS and TPD-MS show that alkali promotion had a beneficial effect on the C–C bond scission of the intermediate acetate. However, the electronic effect of alkali on the structure of Pt metal is not well understood. Different hypotheses can be formulated: (a) charge transfer, which may result in a change in the white line intensity in the presence of K [65]; (b) an electrostatic effect, which could cause bond weakening in adsorbed species on the catalyst surface [66]; (c) a Fermi level electronic perturbation [43]; or (d) an alteration in bond strengths due to changes in the acidity/basicity of the catalyst. Confounding the analysis of (a) is particle size effects [67,68], which tend to cause the binding energy and white line to both increase with decreasing particle size. To gain insight into whether K or Rb promotion leads to charge transfer from the alkali to Pt, the Pt L-3 XANES spectra can be analyzed. As shown in Figure 14 for the K-series and Figure 15 for the Rb-series, during reduction in hydrogen at 350 °C and after cooling in hydrogen to ambient conditions, it is evident that there are differences in the XANES spectra of catalysts having no or low alkali content versus those with high alkali content. However, the white line intensity is also affected by the size of Pt particles, so the difference between the L-3 XANES and L-2 XANES can be studied to remove this effect (Figure 14 and Figure 15). If K or Rb promotion causes electron charge transfer from the alkali to Pt, then the Pt L_3_-L_2_ XANES difference should decrease in magnitude as a function of alkali loading. However, this trend is not observed, indicating that neither potassium nor rubidium is likely transferring electron charge density to platinum. Despite this, it is still suggested that the alkali and Pt are in direct contact, which can be seen through the TPR-XANES and TPR-EXAFS reported in our previous studies [48,49]. The TPR-XANES and TPR-EXAFS revealed that as the loading of K or Rb increases, the reduction of PtO is hindered, which is likely the result of the covering of platinum by the alkali. It is also expected that if the alkali donated electron density to Pt particles, a relaxation in the edge energy should occur (related to the binding energy) [69,70]. Figure 16 and Figure 17 reveal that in comparing catalysts with the Pt^0^ foil, no such shift was detected.

The catalytic activity data for K and Rb-series are reported in Table 3, whereas the H_2_ yield and product selectivity trends of the unpromoted catalyst at different conversions are reported in Table 4. The addition of potassium or rubidium progressively decreased the ethanol conversion. A similar trend was observed for sodium in our previous work [41]. Catalysts with very high alkali loading (i.e., 4.25% K or 9.29% Rb) exhibited negligible catalytic activity. TEM-EDX and EXAFS fittings showed that Pt clusters aggregated at higher loading. Moreover, the alkali likely partially covers the platinum particles, as evidenced by a decreasing ν(CO) band at higher K or Rb alkali loading (virtually disappearing at the highest loadings). However, the most interesting effect of alkali promotion on Pt/ZrO_2_ is related to product selectivity. Indeed, CO is only detected among the products for unpromoted and low alkali doping (0.85% K and 0.93% Rb), whereas no CO is produced at higher alkali loading. Alkali promotion decreased the acetaldehyde selectivity, which is 3.49% for the unpromoted, whereas it is lower than 1.5% for the K- or Rb-promoted catalyst. The activity results confirm that different pathways occur depending on the potassium or rubidium loading, as already pointed out by DRIFTS. The decarbonylation route is present for the unpromoted catalyst and the catalysts with low alkali loading, whereas the decarboxylation route (where acetate decomposes to CH_4_ and a carbonate species, which further decomposes to CO_2_) completely dominates when the alkali loadings reach 2.55% K and 4.93% Rb; a similar effect occurred at 1.80% Na in our prior work [41]. Moreover, 2.55% K corresponds to 5.57% Rb and 1.50% Na on an atomic loading basis. Therefore, the results suggest that decarboxylation is improved by increasing the basicity of the alkali (moving down the Group 1 column) because the atomic loading of potassium to stave off decarbonylation is lower than that of sodium and higher than that of rubidium.

However, Table 4 shows that the product selectivity of Pt/ZrO_2_ changes to a degree as a function of conversion. Therefore, in order to place alkali effects on a firmer footing, it is necessary to compare the product selectivity and H_2_ yield at the same conversion level. As shown in Table 5, the alkali-promoted catalysts have approximately 60–62% higher H_2_ yield as compared to the unpromoted catalyst when compared at similar ethanol conversion. This is due in part to enhanced decarboxylation over decarbonylation, as the CO_2_ selectivity is increased by 240–273%, while the CO selectivity is diminished by 45–61%. While the 0.85% K and 0.93% Rb catalysts provide similar H_2_ yields, 0.85% K corresponds to 1.86% Rb. The fact that a similar improvement in H_2_ yield occurred over that of the unpromoted 2% Pt/ZrO_2_ catalyst at an atomic loading of Rb that is 50% of the atomic loading of K indicates that higher basicity alkali promoters are more effective at facilitating the more selective decarboxylation pathway.

## 4. Conclusions

The addition of potassium or rubidium to Pt/ZrO_2_ progressively decreased the surface area and the pore volume because of some pore blocking. Platinum particle size was ~1 nm for the unpromoted and lower alkali loading (0.85% K and 0.93% Rb), while aggregation occurred at higher alkali loading. TEM-EDX of 9.29% Rb-Pt/ZrO_2_ showed Pt aggregates of 10 nm. The difference between Pt L_3_–L_2_ XANES spectra indicates that neither potassium nor rubidium is likely transferring electron charge density to platinum. Moreover, no relaxation effect on the edge jump energy was observed with the addition of K or Rb.

DRIFTS experiments were carried out to investigate the mechanism. The results suggest that the catalysts have similar steps during ESR: dissociation of ethanol to produce an ethoxy species, oxidative dehydrogenation of ethoxy species to acetate, and acetate decomposition. The forward decomposition of acetate to CH_4_ and carbonate (the precursor to CO_2_) is facilitated by the presence of K or Rb, and there is an optimum alkali loading for facilitating the C–C scission of acetate. This is inferred from the temperature at which CH_4_ evolution occurs as well as a systematic increase in the difference in band position for ν (OCO) asymmetric and symmetric stretching for acetate, which occurs with increasing the alkali loading. Ethoxy species are more stable on the unpromoted catalyst and acetate decomposition, which is associated with the formation of methane, occurs at a higher temperature. Methane formation was detected at 391 °C for 2% Pt/ZrO_2_ in TPD-MS, whereas it occurred at 270 °C for 2.55% K and in multiple peaks (160–180 °C and 370 °C) for 2.79–4.65% Rb. Moreover, DRIFTS experiments and catalytic activity testing point out the existence of different pathways for acetate decomposition depending on the alkali loading. Decarboxylation is the most favored route at high alkali loading (2.55 and 4.25 wt.%). In this pathway, acetate decomposes in the forward direction, yielding CH_4_ and a carbonate species, which further decomposes to CO_2_. In contrast, the unselective decarbonylation pathway occurs to a significant extent for the unpromoted catalyst and the catalyst having low alkali loading (e.g., 0.85% K or 0.93% Rb). By increasing the alkali basicity by switching from K to Rb, lower loadings of alkali enabled virtually complete blocking of the non-selective decarbonylation pathway. Results of unpromoted, K-promoted, and Rb-promoted catalysts compared at similar conversion further confirmed the promoting effect of the alkali, as well as the basicity trend.

## Figures and Tables

**Table 1:** Surface area, porosity, and Pt particle size for K and Rb-promoted catalysts (adapted from [48,49] with permission from Elsevier (copyright, 2020) and MDPI (copyright, 2021)).

Sample ID	A_s_ (BET) (m^2^/g)	V_p_ (BJH Des) (cm^3^/g)	D_p_ (BJH Des) (Å)	Est. Pt Diam (nm)	Est. % Pt Disp. (%)
Pt/ZrO_2_ (K-series)	82.8	0.276	103	1.0 */0.92 **	88
0.85% K-Pt/ZrO_2_	78.3	0.260	101	1.2 */1.1 **	82
1.70% K-Pt/ZrO_2_	72.2	0.249	100	1.3 */1.2 **	79
2.55% K-Pt/ZrO_2_	68.4	0.245	103	2.7 */2.6 **	47
3.40% K-Pt/ZrO_2_	59.6	0.219	105	3.0 */3.0 **	42
4.25% K-Pt/ZrO_2_	53.8	0.200	109	3.6 */3.6 **	35
8.50% K-Pt/ZrO_2_	34.7	0.139	123	-	-
Pt/ZrO_2_ (Rb-series)	89.7	0.260	95	0.8 */0.72 **	94
0.55% Rb-Pt/ZrO_2_	87.9	0.268	96	-	-
0.93% Rb-Pt/ZrO_2_	91.6	0.275	93	0.86 */0.78 **	92
1.86% Rb-Pt/ZrO_2_	88.7	0.262	94	1.0 */0.93 **	87
2.79% Rb-Pt/ZrO_2_	86.7	0.260	93	1.1 */0.99 **	85
4.65% Rb-Pt/ZrO_2_	72.3	0.235	95	1.3 */1.2 **	77
9.29% Rb-Pt/ZrO_2_	58.2	0.202	102	2.0 */1.9 **	56

* Estimated from Jentys assuming spherical cluster morphology [50]. ** Estimated from Marinkovic et al. [51].

**Table 2:** Main bands in cm^−1^ observed for unpromoted, K-promoted, and Rb-promoted 2% Pt/ZrO_2_ catalysts.

Bands	0% K	0.85% K	2.55% K	4.25% K	0% Rb	0.93% Rb	4.65% Rb	9.29% Rb
**50 °C**								
ν(CO) ethoxy	1100, 1070, 1056	1092, (1065), 1051	1103, 1058	1107, (1067) 1056	1101, 1072, 1057	1099, (1067), 1055	1098, 1056	1105, 1057
ν(CH) ethox/acet	2970, 2928, 2897, 2868	2970, 2927, (2894), 2872	2969, 2926, (2897), 2876	(2977), 2965, (2934, 2881), 2868	2973, 2929, (2896), 2873, (2854)	2973, 2929, (2898), 2873, (2856)	2973, 2931, 2898, 2879, 2858	(2989), 2970, (2955), 2932, 2902, 2880, 2868
ρ(CH_3_) ethoxy	1156, 1116	1148, 1124	(1147, 1125)	-	1154, (1133), 1118	(1163, 1150, 1128–1113)	-	-
ν_a_(OCO) acetate	1562	1564, (1507)	1577, (1519)	1567–1513	1564	1560	1572, (1549–1512)	1578
ν_s_(OCO) acetate	(1467), 1433	1467, (1431), 1417	(1490, 1464) 1412	1460	(1470), 1433	(1487) 1474, 1443, 1428	1472, (1445), 1408	-
δ_s_(CH_3_) acetate	1381, (1357), 1344, (1274)	1376, (1351) 1335, (1314–1269)	1377, 1358, (1340, 1280)	1358, 1310, 1271	1381, (1358, 1343, 1276)	1381, 1357, 1343, 1327–1295, 1275	(1372), 1360, (1339)	1355
ν(CO) Pt-CO	2055, (2037), 2012, 1990–1810	2051, 2030, (2018–1870)	1930 (2080–1800)	-	2051, (2032), 2015, 1978–1870	(2053, 2046–1985, 1985–1840), 1951	(2063–1985, 1963), 1927, (1950–1830)	-
**200 °C**								
ν_a_(OCO) acetate	**1556**, (1470)	(1569), **1557**, (1543–1508), 1467	**1580**, (1549, 1523, 1508), 1471	**1580**, (1550–1444)	(1566), **1554**, (1470)	**1556**, (1525–1470)	1633, **1579**, (1549, 1533–1465)	**1580**
ν_s_(OCO) acetate	**1439**	**1437**, 1380	**1408**	1422, **1408**	**1439**	**1437**, (1383)	**1404**	1437, **1404**
δ_s_(CH_3_) acetate	1346	1332, (1303), 1273	(1351), 1334, (1297)	(1350), (1330)	1347	1339, (1330-1312, 1297, 1274)	(1328), 1300	1355, 1339, (1335–1267)
ν(CH) acetate	2965, **2937**, 2876, 2862	3000, 2984, 2966, **2931**, 2872	2971, **2928**, (2897, 2878, 2858)	(2965), 2937, 2869	2965, **2936**, (2917–2892), 2877	2965, **2937**, (2920–2905, 2881	2986, 2967, **2927**	(2997), 2965, (2935), 2925, (2904, 2886–2860)
**500 °C**								
ν_a_(OCO) carbonate	1556	1622, 1576–1490	1620, (1568–1492)	(1604), 1551, (1530, 1519, 1503)	1550–1500	1580–1495, 1556	1627, (1581), (1550–1430)	1608, (1592, 1566, 1550)
ν_s_(OCO) carbonate	(1470), 1439	1454	(1468)	(1463, 1445, 1425)	1473, 1442	1490-1410, 1444	(1550–1430)	1439
ν_s_(OCO) carbonate	(1405–1367)	1370, (1342) 1297, (1276–1225)	(1386, 1353) 1307	1400-1355, 1329, (1300–1200)	1395, 1363	(1373), 1340, (1300), 1271, (1256)	(1365, 1333, 1291)	(1355), 1317

**Table 3:** ESR catalytic activity for K-series and Rb-series (300 °C, 1 atm, 190,560 Ncc/h/g_cat_, feed: C_2_H_5_OH 2.98% H_2_O 26.14% N_2_ 70.88%).

Catalyst	Conv. C_2_H_5_OH (%)	H_2_ Yield (%)	C Selectivity (%)						
			CH_4_	CO_2_	CO	C_2_H_6_	C_2_H_4_	C_3_H_6_	CH_3_CHO
2% Pt/ZrO_2_	86.91	14.26	45.20	28.5	21.16	0.92	0.39	0.34	3.49
0.85% K-2% Pt/ZrO_2_	60.01	13.84	46.86	40.59	11.14	0.26	-	-	1.14
2.55% K-2% Pt/ZrO_2_	47.02	12.66	48.75	50.72	-	-	-	-	0.76
4.55% K-2% Pt/ZrO_2_	27.30	3.17	49.48	49.08	-	-	-	-	1.42
0.93% Rb-2% Pt/ ZrO_2_	59.60	13.65	46.81	35.78	15.55	0.31	0.11	-	1.43
4.25% Rb-2% Pt/ZrO_2_	38.97	8.70	53.56	46.27	-	-	-	-	0.16
9.29% Rb-2% Pt/ZrO_2_	11.19	0.18	12.18	87.81	-	-	-	-	-

**Table 4:** ESR catalytic activity for 2% Pt/ZrO_2_ catalyst at different C_2_H_5_OH conversion (300 °C, 1 atm, feed: C_2_H_5_OH 2.98% H_2_O 26.14% N_2_ 70.88%).

Catalyst	Conv. C_2_H_5_OH (%)	H_2_ Yield (%)	C Selectivity (%)						
			CH_4_	CO_2_	CO	C_2_H_6_	C_2_H_4_	C_3_H_6_	CH_3_CHO
2% Pt/ZrO_2_	86.91	14.26	45.20	28.5	21.16	0.92	0.39	0.34	3.49
	58.55	8.53	47.46	14.86	28.37	0.37	0.57	0.36	7.98
	48.41	4.81	55.87	11.56	22.07	0.24	0.49	-	9.73
	30.89	3.05	60.79	7.13	19.70	-	0.48	-	11.81

**Table 5:** ESR catalytic activity for select catalysts at the same C_2_H_5_OH conversion (300 °C, 1 atm, feed: C_2_H_5_OH 2.98% H_2_O 26.14% N_2_ 70.88%) for selectivity comparison.

Catalyst	Conv. C_2_H_5_OH (%)	H_2_ Yield (%)	C Selectivity (%)						
			CH_4_	CO_2_	CO	C_2_H_6_	C_2_H_4_	C_3_H_6_	CH_3_CHO
2% Pt/ZrO_2_	58.55	8.53	47.46	14.86	28.37	0.37	0.57	0.36	7.98
0.85% K-2% Pt/ZrO_2_	60.01	13.84	46.86	40.59	11.14	0.26	-	-	1.14
0.93% Rb-2% Pt/ ZrO_2_	59.60	13.65	46.81	35.78	15.55	0.31	0.11	-	1.43

## Data Availability

Not applicable.
